# Painless Aortic Dissection—Diagnostic Dilemma With Fatal Outcomes: What Do We Learn?

**DOI:** 10.1177/2324709617721252

**Published:** 2017-07-31

**Authors:** Saeeda Fatima, Konika Sharma

**Affiliations:** 1 Bassett Medical Center, Cooperstown, NY, USA

**Keywords:** painless aortic dissection, atypical acute aortic dissection

## Abstract

Aortic dissection is the most catastrophic clinical condition that involves the aorta. It has a high mortality as well as high rate of misdiagnosis due to frequent unusual presentation. Typically, it presents with acute chest, back, and tearing abdominal pain. However, it can present atypically with minimal or no pain, making diagnosis difficult. Physicians should always suspect acute aortic dissection in patients with certain clinical conditions like difficult-to-control hypertension, giant cell arteritis, bicuspid aortic valve, intracranial aneurysms, simple renal cysts, family history of aortic disease, and Marfan syndrome, especially when a patient presents with ischemic symptoms involving multiple organ without an obvious cause.

## Introduction

Aortic dissection is the most catastrophic clinical condition that involves the aorta. It has complex clinical manifestations, and consequently, it has a high delayed and missed diagnosis rate. Typically, it presents with acute chest, back, and tearing abdominal pain. However, it can present atypically with minimal or no pain, making diagnosis difficult.^1^ We will discuss 2 cases of fatal type A acute aortic dissection (AAD) whose initial presentations were atypical, presenting as multi-organ failure, gastrointestinal bleed, and transient ischemic attack–like symptoms.

## Case 1

**Figure 1.**
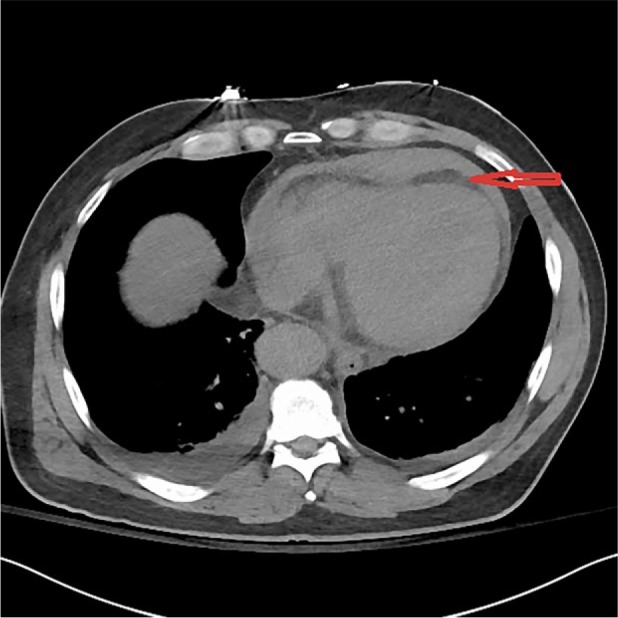
Arrow showing pericardial effusion on non-contrast CT. No dissection flap is seen.

**Figure 2.**
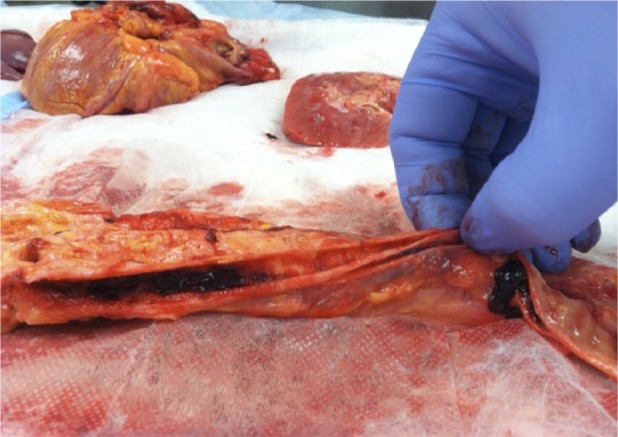
Autopsy specimen showing dissection of aorta.

A 52-year-old male was transferred to our facility from an outside emergency department (ED) where he presented with a constellation of symptoms including extreme fatigue and diaphoresis for 12 hours, dyspepsia, and an episode of vomiting, black tarry stools, and syncope. His history was significant for hypertension, alcohol abuse, hypothyroidism, and hyperlipidemia, and he was recovering from a recent toe infection requiring surgery. Family history was noncontributory. He was an everyday smoker with a greater than 20-pack-year history. On review of systems, he had no chest or back pain, shortness of breath, headache, visual changes, or focal weakness. In the outside hospital, his blood pressure (BP) was 136/102 mm Hg, heart rate (HR) 88/min, respiratory rate (RR) 18/min, and was afebrile. Initial laboratory tests in the ED showed leukocytosis (15 000 cells/mm^3^), lactate 8.2 mmol/L, international normalized ratio 1.5, aspartate aminotransferase (AST) 122 U/L, creatinine 2.6 mg/dL, blood urea nitrogen (BUN) 25 mg/dL, and troponin elevation 0.3 ng/mL. Electrocardiogram (EKG) revealed normal rate and rhythm and no ischemic changes. Chest X-ray did not show any infiltrate or mediastinal widening. Given the patient’s recent toe surgery, the emergency physician diagnosed him as sepsis with multi-organ failure and treated him with broad-spectrum antibiotics including vancomycin and piperacillin/tazobactam, intravenous fluids (IV), and transferred the patient to our facility. On arrival to the hospital, the patient had high BP 180/110 mm Hg, HR 86/min, RR 18/min . Significant physical exam findings included grade II systolic murmur heard in the left sternal border, absent left dorsalis pedis, popliteal, and femoral pulses but no cyanosis, and bright red blood on digital rectal exam. Repeat labs at 24 hours from onset of symptoms again showed leukocytosis, persistently elevated lactate 8.3 mmol/L, worsening liver enzymes (AST 1127 U/L, alanine transaminase [ALT] 988 U/L), worsening kidney function (creatinine 2.9 mg/dL, BUN 28 mg/dL), elevation in troponin 1.0 ng/mL, and elevated lactate dehydrogenase (LDH) 1863 U/L. The patient remained oliguric since initial presentation despite IV fluids. Non-contrast computerized tomography (CT) showed pericardial effusion, peripancreatic fluid and fluid in colonic gutters, diverticulosis, urinary bladder hematoma (Figure 1). Transthoracic echocardiogram showed low ejection fraction of 35%, dilated aortic root, and a moderate pericardial effusion with impending tamponade. It remained a diagnostic dilemma for the medical team to piece together all the aforementioned signs and symptoms. Multiple subspecialties were involved including critical care, cardiology, nephrology, gastroenterology, urology, and hematology. At 48 hours, while the patient’s condition remained a puzzle, he went into pulseless arrest. Despite 45 minutes of resuscitative efforts, the patient could not be revived. Autopsy revealed dissection of aorta extending from aortic root to the superior mesenteric artery and rupture into the pericardial space (Figure 2).

## Case 2

**Figure 3.**
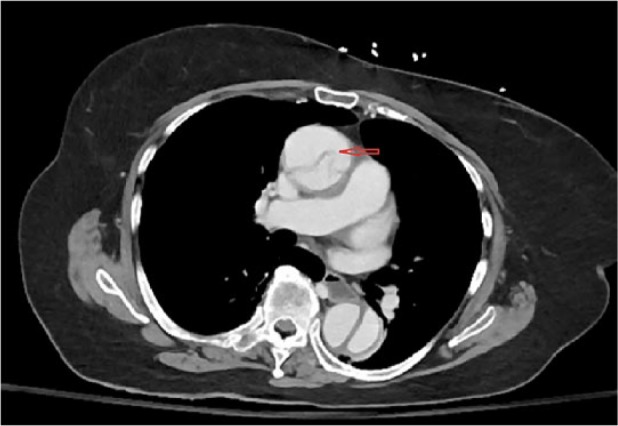
Arrow indicating to dissection flap in the ascending aorta on the contrast enhanced CT.

**Figure 4.**
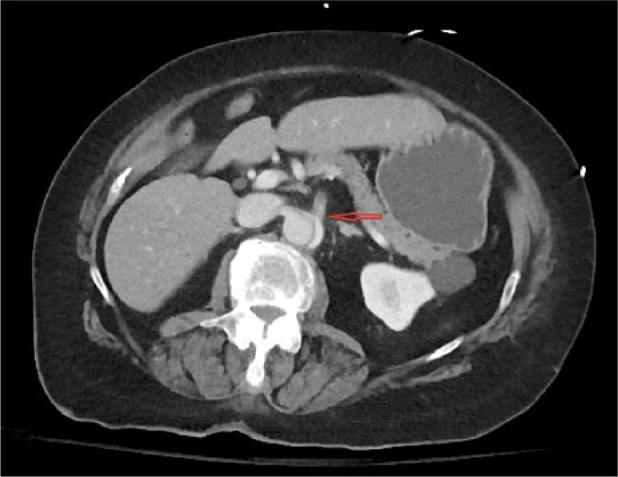
Arrow indicating to dissection flap extending to superior mesenteric artery (SMA) on contrast enhanced CT.

A 78-year-old woman presented to the ED with complaints of nausea, vomiting, anorexia, and generalized weakness for 6 hours. These symptoms were preceded by a transient left jaw pain and left arm and leg weakness for a few seconds. She also complained of mild dull achy abdominal discomfort of a few hours’ duration. On arrival to the ED, she had another episode of vomiting and 2 bloody bowel movements. Her medical history was significant for duodenal ulcer, hypertension, deep venous thrombosis, diabetes, and giant cell arteritis (GCA). Family history was significant for late-onset coronary artery disease in both parents. She had a remote 4-pack-year smoking history. On examination, BP was 136/60 mm Hg, HR 60/min, RR 20/min, and SpO_2_ 96% on room air. Pulses were equal in both arms and legs. Abdomen was nontender and rectal exam was positive for bright red blood. Detailed neuro exam failed to show any focal signs. Laboratory test showed leukocytosis 18.1 cells/mm^3^, lipase 598 U/L, AST 56 U/L, and LDH 344 U/L. Troponin was negative, and creatinine, BUN, and ALT were within normal limits. Other ED workup included unremarkable CT head and chest X-ray with no focal findings or mediastinal widening. At 9-hour time point from symptom onset, the patient was admitted to medical service. The neurological symptoms were considered to be manifestations of a transient ischemic attack given rapid resolution of symptoms. Diagnostic focus was shifted to hematochezia and belly discomfort, considering it to be an ischemic colitis. At 11 hours, the patient underwent a CT abdomen with oral and intravenous contrast. Just after the imaging was finished, the patient suddenly became unresponsive. Resuscitative efforts and advance life support were not initiated because she had already signed advance directives of no resuscitative efforts. CT scan revealed dissection of aorta extending from aortic root to superior mesenteric artery and left common iliac artery (Figure 3) (Figure 4). Before the findings were communicated, the patient passed away.

## Discussion

Acute aortic dissection is a medical emergency and has a high mortality as well as high rate of misdiagnosis due to frequent atypical presentation. According to Stanford classification, type A dissection involves the ascending aorta and may progress to involve the arch and thoracoabdominal aorta. Type B dissection involves the descending thoracic or thoracoabdominal aorta distal to the left subclavian artery without involvement of ascending aorta. Some of the risk factors and comorbid conditions associated with both type A and type B dissection are shown in table 1.^2-4^ In the International Registry for Aortic Dissection (IRAD), severe chest pain remains the most common presenting feature in both type A and type B AAD. However, painless AAD is more prevalent among patients suffering from type A dissection than type B dissection and is associated with increased mortality.^5^ About 6.4% of AADs are painless. Other common features of type A AAD are hypotension, pulse deficits, and mediastinal widening.^1,5,6^ Our first case had difficult-to-control hypertension and was on multiple BP controlling pills. He did have a pulse deficit which was neglected in the conundrum of clinical presentations and the physicians were unable to connect the dots in a unifying diagnosis. On retrospective evaluation of the case and independent review by two radiologists, no flap was seen on non-contrast CT. However, neglecting the absence of distal pulses was a mistake on part of the medical team and shouldn’t have been overlooked. If physicians had put together absent pulses along with visceral ischemia and considered a trans-esophageal echocardiogram, a timely diagnosis of dissection could have been made. In the second case, the presentation was again very misleading, but the clue to diagnosis would have been history of GCA, which was overlooked as she has no other typical features of aortic dissection on initial examination and objective data. The patient developed a rapid decline over a period of 11 hours and sudden cardiac arrest was likely due to extension of dissection resulting in cardiac tamponade. AAD is more common in females with history of GCA and is not usually related to size of aneurysm.^7^ Interestingly, a CT scan of her chest done 2 years ago did not reveal aneurysm.

**Table 1.** Common Risk Factors for Aortic Dissection.

● Long-standing hypertension
● Smoking and cocaine use
● Connective tissue disorder (eg, Marfan syndrome, Ehlers Danlos syndrome)
● Bicuspid aortic valve
● Coarctation of aorta
● Hereditary thoracic aneurysm
● Intracranial aneurysms
● Simple renal cysts
● Vascular inflammation (eg, GCA, Takayasu arteritis)
● Syphilis
● Deceleration trauma

In the IRAD study, the common presentations in painless dissection include syncope (33.9%), new-onset neurological deficit (23.7%), stroke, congestive heart failure (19.7%), coma or spinal cord ischemia (17.0%), acute renal failure (13.6%), myocardial infarction (7.1%), and mesenteric ischemia or infarction (6.8%).^1^ Diagnosis of AAD requires a high degree of clinical suspicion and should always be considered by physicians in patients with the above-mentioned features where exact diagnosis is uncertain.^8^ Chest X-ray and EKG are frequently ordered tests in the ED but results are often equivocal. In recent years, the reported incidence of normal chest X-rays among patients with AAD has increased. Furthermore, the finding of a widened mediastinum on chest X-rays of IRAD patients has been less frequent than historic norms and has been found in only 54.3% of type A patients.^5^ No mediastinal widening was seen in either of the aforementioned cases. D-dimer is also one of the nonspecific tests, and the cutoff value is the same as used for pulmonary embolism. Transthoracic echocardiography can sometimes diagnose dissection. A small prospective cohort study showed that in patients presenting in shock, transthoracic echocardiography had a 78.3% sensitivity and 83.0% specificity for diagnosing proximal dissection.^9^ Transesophageal echocardiography has high sensitivity and specificity, but it is operator dependent and is not always available. CT has been increasingly used in recent years and helps clinicians to rapidly confirm or exclude aortic dissection.^9,10^

## Conclusion

Our case series reinstates a few important learning points. First, it is important to consider all the relevant past and present information to look for important clues in order to develop a right differential diagnosis in challenging clinical presentations. Although this seems very obvious, sometimes clinicians tend to neglect important information because of anchoring and heuristic bias, which leads to medical errors with critical implications. Secondly, when patients present with signs and symptoms of ischemic injury affecting multiple organs without an obvious cause, aortic dissection should be considered, even without the presence of a characteristic pain as supported by the literature and evidence mentioned above.

## References

[1] Pape LA Awais M Woznicki EM Presentation, diagnosis, and outcomes of acute aortic dissection: 17-year trends from the International Registry of Acute Aortic Dissection. J Am Coll Cardiol. 2015;66:350-358. 26205591 10.1016/j.jacc.2015.05.029

[2] Ziganshin BA Elefteriades JA Guilt by association: a paradigm for detection of silent aortic disease. Ann Cardiothorac Surg. 2016;5:174-187. 27386404 10.21037/acs.2016.05.13 PMC4893529

[3] Nienaber CA Eagle KA Aortic dissection: new frontiers in diagnosis and management. Circulation. 2003; 108(5):628-635. 12900496 10.1161/01.CIR.0000087009.16755.E4

[4] Tsai TT Nienaber CA Eagle KA Acute aortic syndromes. Circulation. 2005;112(24):3802-3813. 16344407 10.1161/CIRCULATIONAHA.105.534198

[5] Park SW Hutchison S Mehta RH Association of painless acute aortic dissection with increased mortality. Mayo Clin Proc. 2004;79:1252-1257. 15473405 10.4065/79.10.1252

[6] Liu Z Zou Y Chai B Zeng H Analysis of clinical features of painless aortic dissection. J Huazhong Univ Sci Technolog Med Sci. 2014;34:582-585. 25135731 10.1007/s11596-014-1319-8

[7] Nayar AK Casciello M Slim JN Slim AM Fatal aortic dissection in a patient with giant cell arteritis: a case report and review of the literature. Case Rep Vasc Med. 2013;2013:e590721. 10.1155/2013/590721 PMC3600339 23533937

[8] Demircan A Aksay E Ergin M Bildik F Keles A Aygencel G Painless aortic dissection presenting with acute ischaemic stroke and multiple organ failure. Emerg Med Australas. 2011;23:215-216. doi:10.1111/j.1742-6723.2011.01389. 21489170

[9] Thrumurthy SG Karthikesalingam A Patterson BO Holt PJE Thompson MM The diagnosis and management of aortic dissection. BMJ. 2012;344:d8290. 10.1136/bmj.d8290 22236596

[10] Imamura H Sekiguchi Y Iwashita T Painless acute aortic dissection. Diagnostic, prognostic and clinical implications. Circ J. 2011;75:59-66. 21099124 10.1253/circj.cj-10-0183

